# A new *GTF2I-BRAF* fusion mediating MAPK pathway activation in pilocytic astrocytoma

**DOI:** 10.1371/journal.pone.0175638

**Published:** 2017-04-27

**Authors:** Tajana Tešan Tomić, Josefin Olausson, Annica Wilzén, Magnus Sabel, Katarina Truvé, Helene Sjögren, Sándor Dósa, Magnus Tisell, Birgitta Lannering, Fredrik Enlund, Tommy Martinsson, Pierre Åman, Frida Abel

**Affiliations:** 1 Department of Clinical Genetics, Institute of Biomedicine, Sahlgrenska Academy at the University of Gothenburg, Gothenburg, Sweden; 2 Children´s Cancer Centre, The Queen Silvia Children's Hospital, Gothenburg, Sweden; 3 Bioinformatics core facility, Sahlgrenska academy, University of Gothenburg, Gothenburg, Sweden; 4 Department of Clinical chemistry, Sahlgrenska University Hospital, Gothenburg, Sweden; 5 Department of Pathology, Sahlgrenska University Hospital, Gothenburg, Sweden; 6 Department of Neurosurgery, Sahlgrenska University hospital, Gothenburg, Sweden; 7 Sahlgrenska Cancer Center, Institute of Biomedicine, Sahlgrenska Academy at the University of Gothenburg, Gothenburg, Sweden; University of Michigan, UNITED STATES

## Abstract

Pilocytic astrocytoma (PA) is the most common pediatric brain tumor. A recurrent feature of PA is deregulation of the mitogen activated protein kinase (MAPK) pathway most often through *KIAA1549-BRAF* fusion, but also by other *BRAF*- or *RAF1*-gene fusions and point mutations (*e*.*g*. *BRAF*V600E). These features may serve as diagnostic and prognostic markers, and also facilitate development of targeted therapy. The aims of this study were to characterize the genetic alterations underlying the development of PA in six tumor cases, and evaluate methods for fusion oncogene detection. Using a combined analysis of RNA sequencing and copy number variation data we identified a new *BRAF* fusion involving the 5’ gene fusion partner *GTF2I* (7q11.23), not previously described in PA. The new *GTF2I-BRAF* 19–10 fusion was found in one case, while the other five cases harbored the frequent *KIAA1549-BRAF* 16–9 fusion gene. Similar to other *BRAF* fusions, the *GTF2I-BRAF* fusion retains an intact *BRAF* kinase domain while the inhibitory N-terminal domain is lost. Functional studies on *GTF2I-BRAF* showed elevated MAPK pathway activation compared to *BRAF*^*WT*^. Comparing fusion detection methods, we found Fluorescence in situ hybridization with *BRAF* break apart probe as the most sensitive method for detection of different *BRAF* rearrangements (*GTF2I-BRAF* and *KIAA1549-BRAF*). Our finding of a new *BRAF* fusion in PA further emphasis the important role of B-Raf in tumorigenesis of these tumor types. Moreover, the consistency and growing list of *BRAF/RAF* gene fusions suggests these rearrangements to be informative tumor markers in molecular diagnostics, which could guide future treatment strategies.

## Introduction

Central nervous system (CNS) tumors are the second most common pediatric malignancies after acute lymphoblastic leukemia. Among all brain tumors, low-grade gliomas (LGG, World Health Organization (WHO) grade I and grade II) account for around 30–40% of cases [1]. The most common LGGs are the Pilocytic astrocytomas (PA, grade I) accounting for at least 17% of CNS neoplasms in children (0–14 years) [2]. The majority of pediatric PA occurs in the cerebellum (>40%), but can also be found in the supratentorial compartment, the optic pathway, hypothalamus, brainstem and spinal cord [3]. PA are histologically characterized by bipolar tumor cells, biphasic pattern, Rosenthal fibers and eosinophilic granular bodies but can exhibit varying histology and can show similarities to other high-grade astrocytomas, making the diagnosis somewhat challenging [4, 5]. PA has a favorable prognosis indicated by 20 years survival rate of 90% for low-grade astrocytomas [1]. Dissemination is uncommon, but may occur in newly diagnosed PAs [2]. Surgical resection is a first line therapy, and radiation and chemotherapy are applicable in case of inoperable or partly resected tumors. Despite good prognosis, recurrence of the tumor occurs in 10–20% of cases and the effects of tumor and current treatment strategies can cause severe psychosocial and physical dysfunction [6]. This emphasizes considerable need for reliable tumor markers to improve histological diagnosis of PA and ensure appropriate therapy, but also to guide and facilitate the development of personalized targeted therapy.

Until recently, the molecular mechanisms involved in development of PA were largely unknown. Through large genome wide DNA copy number variation (CNV) studies, gene fusions involving *RAF* paralogs were identified in PA [7–9]. These fusions are formed by tandem duplications or deletions on chromosome arms 7q.34 (involving *BRAF*) [7, 9–11] and 3p (involving the less common *RAF1* gene) [8, 12]. Today, the *KIAA1549-BRAF* fusion, is the most prevalent genetic alteration in pediatric PA accounting for around 90% of cases [7]. Currently, there are several known *KIAA1549-BRAF* fusion junctions, where *KIAA1549-BRAF* 16–9 (∼60%); 15–9 (∼30%); 16-11(∼10%) fusions are the most prevalent ones, whereas *KIAA1549-BRAF* 18–10, 19–9, 16–10, 15–11, 17–10 fusions are more rare (< 1%) [7–9, 13, 14]. Also, other less frequent gene fusions found in PAs are *FAM131-BRAF*, *SRGAP3-RAF1*, *RNF130-BRAF*, *CLCN6-BRAF*, *MKRN1-BRAF* and *GNAI1-BRAF* [10, 12, 15], and the list of new *RAF/BRAF* fusions is continuously growing. The common feature for all reported *BRAF/RAF* fusions is the absence of inhibitory N-domain leading to constitutive active RAF kinase [7, 10, 12, 16]. In addition to gene fusions, point mutations in the MAPK pathway (*e*.*g*. *BRAF*, *FGFR1*, *NF1*, *KRAS*) can be found in PA, although rare [15]. Point mutations and rearrangements are reported to be mutually exclusive in this tumor type [15], which highlight a central role of the MAPK pathway in tumorigenesis.

The presence of the *KIAA1549-BRAF* fusion is associated with improved outcome in PA, and has been suggested as a prognostic marker [17]. However, it still remains generally accepted that patient age, location of the tumor, and extent of resection are the strongest prognostic indicators [18]. Since the *KIAA1549-BRAF* fusions are highly prevalent in pediatric PA, this feature can be used as a supportive diagnostic marker in cases where neuropathological distinction from other gliomas is difficult [19, 20]. The diagnostic and prognostic potential of *KIAA1549-BRAF* fusion in addition to ongoing development and evaluation of MAPK pathway targeted therapy requires reliable detection of all *BRAF* rearrangements for correct molecular subgrouping of tumors and patients treatment groups. To date, several different methods are used for molecular characterization of *BRAF/RAF*-rearrangements, *e*.*g*. quantitative PCR (qPCR), Fluorescence in situ hybridization (FISH), Copy number variation (CNV) microarray, and RNA sequencing, and there is no consensus regarding best practices for *BRAF/RAF*-fusion detection.

The aim of this study was to search for fusion oncogenes in a set of six pediatric PA and to evaluate methods for detection of *BRAF* aberrations. Through combined RNA sequencing and CNV detection we discovered a new *GTF2I-BRAF* 19–10 gene fusion in one PA case, which displayed MAPK activating properties. The four fusion-detection methods evaluated in this paper suggest the FISH break apart probe for *BRAF* to be the most suitable method for detection of different kinds *BRAF* rearrangement, irrespectively of its exon junction or fusion partner.

## Material and methods

### Patient data

Six PA tumors were collected from pediatric patients (1–18 years) that underwent surgical resection between years 2000–2003 at the department of Neurosurgery, Sahlgrenska University hospital, Gothenburg, Sweden. Tumor tissue was fresh-frozen at surgery or preserved in RNA-later (Thermo Fisher Scientific, www.thermofisher.com). Patients were followed up at the Children´s Cancer Centre, Queen Silvia Children's Hospital, Sahlgrenska University hospital (Table 1). Diagnosis was made by histological examination by a neuropathologist following the WHO criteria [5] (S1 Fig). The study was approved by the Regional Ethical Review Board in Gothenburg (approved 2013-05-22; approval number: Dnr 239–13). Written informed consent was obtained from the parents, caretakers, or guardians on behalf of the minors/children (<18 years old) enrolled in the study.

**Table 1:** Clinical data of the PA cases.

Sample ID	Sex	Age (years)	Location	Extention of resection	Event	Other Treatment	Status	Follow-up (months)	Last Follow-up (date)
PA1	M	13	C	GTR	Recurrence (local)	Re-op nov-01	AND	121	aug-09
PA2	M	9	C	GTR	No	No	AND	83	aug-07
PA3	F	17	C	GTR	Pituitary prolactinoma	(Dopamine agonist for prolactinoma)	AND	36	nov-03
PA4	F	12	C	GTR	No	No	AND	95	jan-09
PA5	F	8	C	GTR	No	No	AND	45	nov-04
PA6	F	4	C	GTR	No	No	AND	79	jan-09
PA6	F	4	C	GTR	No	No	AND	79	jan-09

Sex: F, female; M, male; Age: Age at diagnosis (years). Location: C, cerebellum; Extention of resection = Partial (PR) or Gross Total resection (GTR). Event = Recurrence or No or Other. Other Treatment = description of other treatment in addition to resection. Re-op = reoperation. AND, Alive no evidence of disease; AWD, alive with disease; D, death.

### DNA/RNA isolation and cDNA synthesis

Genomic DNA (gDNA) and total RNA were extracted from 25-30mg fresh frozen tumor tissue using the Tissue DNA Purification Kit and Simply RNA Tissue Kit respectively, and run on the Maxwell 16 instrument according to manufacturer’s protocol (Promega, Madison, WI, USA). Sample concentration and purity was measured with DS-11 Spectrophotometer (De Novix, http://www.denovix.com) and QuBit fluorometer (Thermo Fisher Scientific). The integrity of total RNA was assessed using a Bioanalyzer (Agilent Technologies, http://www.agilent.se), and RNA Integrity Numbers (RIN) from the six RNA samples were within the range of 6.7–8.6. A total amount of 750 ng total RNA was reverse transcribed into cDNA using SuperScript VILO cDNA Synthesis Kit according to manufacturer’s protocol (Thermo Fisher Scientific).

### Quantitative PCR

Quantitative RT-PCR (qPCR) was performed in 384-well format using the ABI PRISM 7900HT instrument (Applied Biosystems by Life technologies, Thermo Fisher Scientific). TaqMan primers and probes for *KIAA1549-BRAF* fusions were custom-designed based on Tian et al. [21] and ordered directly from Life Technologies (S1 Table). Amplification reactions (10 μl) were carried out with 1 μl of 1:4 diluted template cDNA (approximately 20ng total RNA converted to cDNA), 1 x TaqMan Universal PCR Master Mix (Applied Biosystems), 1 x FAM-labeled Assay-on-Demand Gene expression Assay Mix (Applied Biosystems). Thermal cycling was initiated with a 2 minute incubation at 50°C, followed by a first denaturation step of 10 minutes at 95°C, and then by 40–50 cycles of 15 seconds at 95°C and 1 minute at 60°C. The qPCR amplification results were analyzed in the Sequence Detection System (SDS) software (Applied Biosystems). The *KIAA1549-BRAF* gene fusion status was determined as positive (quantitation cycle (Cq)<30) or negative (undetermined).

### Competitive allele-specific TaqMan PCR

Competitive Allele-Specific TaqMan PCR (castPCR) was performed to detect and measure somatic mutation of *BRAF*V600E using the TaqMan Mutation Detection Assay (Hs00000111_mu, Applied Biosystems) for c.1799T>A in *BRAF* (RefSeq accession no: NM_004333.4). A “B-Raf V600E Genomic DNA Reference Standard” sample (Horizon Diagnostics, https://www.horizondiscovery.com) was used as positive control in a 1:2 dilution series of six samples (corresponding to an allelic frequency range: 0.78–50%), and a blood donor gDNA was used as a negative control. Mutation detection by castPCR was carried out in 10 μL reactions in 384-well format, each well comprising 20ng gDNA template, 1X Genotyping Master Mix, and 1X TaqMan Mutation Detection Assay (containing allele-specific forward primer, locus-specific TaqMan probe, locus-specific reverse primer, allele-specific MGB blocker), and run on an Applied Biosystems QuantStudio Real-Time PCR System using the following thermal cycling conditions: 95°C for 10 minutes; 5 cycles: 92°C for 15 seconds and 58°C for 1 minute; 40 cycles: 92°C for 15 seconds and 60°C for 1 minutes. All samples were run in triplicates. The mutation status and allele frequency (AF) was analyzed using Mutation Detector TM software (Applied Biosystems). Each sample was considered as positive (AF>0.1%) or negative (AF = 0%) for the *BRAF*V600E mutation.

### RNA sequencing and data analysis

One μg of total RNA per sample was used as starting material for high-throughput paired-end 2 x 100 base pairs (bp) RNA sequencing run at an Illumina HiScanSQ instrument (https://www.illumina.com). The library preparation was performed according to manufacturer’s instruction using the TruSeq Stranded Total RNA Sample Preparation Kit (Protocol #15031048 rev C) with an integrated rRNA-depletion step using Ribo-Zero (Nordic biolabs, http://www.nordicbiolabs.se). The rRNA-depletion efficiency was checked by qPCR (with TaqMan) targeting *18S* and *GAPDH*, by comparing the expression before and after removal of rRNA. The cDNA library was size selected, range 200–450 bp, using the PippinPrep instrument according to the manufacturer’s instructions (Sage Science, http://www.sagescience.com). The Illumina software pipeline was used to process image data into raw sequencing data. The quality of the raw sequence data was assessed using FastQC software. (http://www.bioinformatics.babraham.ac.uk/projects/fastqc/), generating a total of 200–300 million reads per sample. To search for fusion transcripts the FusionCatcher algorithm (version FusionCatcher_99.3e_ensembl v.77-May-2015) was run by default settings [22]. The associated ENSEMBL, UCSC, and RefSeq databases were automatically downloaded by FusionCatcher (https://code.google.com/p/fusioncatcher/). The output from FusionCatcher contained preliminary list and one final list of candidate fusion genes per sample. The final list output required two spanning and three supporting reads per fusion.

### Copy number variation profiling

CNV genomic profiling of the six tumors was performed with CytoScan HD arrays (Affymetrix, Inc., Santa Clara, CA) according to manufacture’s protocol. The CytoScan HD array comprises more than 2.67 million copy number markers of which 750 000 are SNP probes and 1.9 million are non- polymorphic probes. Briefly, total genomic DNA (250 ng) was digested (NspI), ligated, PCR amplified, fragmented with DNase I, labeled with biotin and hybridized to a CytoScan HD array for 16–18 hours. The hybridized probes were washed using the GeneChip Fluidics Station 450, and marked with streptavidin-phycoerythrin. The arrays were scanned using a confocal laser scanner, GeneChip Scanner 3000 (Affymetrix, Inc.). Data analysis was performed with Affymetrix Chromosome Analysis Suite (ChAS) version 3.0 (Affymetrix, Inc.). CEL files were analyzed and converted to CYCHP result files by Single Sample Analysis and Normal Diploid Analysis in ChAS. Samples were viewed and inspected in ChAS browser. The calling threshold of CNVs for the CytoScan HD Array was set to the following: segment filter settings ≥ 200 kb with markercount ≥ 50. Manual screening was also performed through a number of parameters given by ChAS, such as smooth signal, weighted log2 ratio, and allele difference.

### Bioinformatics

The stating of coding gene fusions was according to two criteria; 1) called gain/loss in CNV profiles by ChAS, and 2) presence of supporting reads in the RNA-seq data. Hence, the location of coding predicted fusions from the final list by FusionCatcher (S2 Table) were verified by inspection of CNV changes and breakpoints in the ChAS browser. Vice versa, genes located in CNV breakpoint regions from gain/loss segments called by ChAS were verified by their presence in FusionCatcher preliminary and final lists. In addition, for suspect CNV fusion junctions that could not be found in the FusionCatcher output lists, a 30 bp match sequence adjacent to the junction was utilized to screen through the RNA-seq data for supporting spanning reads verifying the breakpoint. The screening was performed using The BLAST-Like Alignment Tool (BLAT) [23] on the sequencing data in fasta format.

To identify all sequence reads around the fusion points, 600bp coding sequences for each potential exon-exon junction, 300 bases upstream (5´-end gene), and 300 bases downstream (3´-end gene) was outlined from mRNA reference sequences (S3 Table). Next, all reads were mapped to these coding sequences using BLAT with default settings. Spanning reads were defined as those spanning the exon-exon junction or fusion breakpoint. Split reads were required to clearly map to both sides of the exon-exon junction or fusion breakpoint with no spanning reads at the breakpoint. To avoid false positives it was required that at least 70 out of 75 bases should map coherently to the reference sequence, therefore the distance from map-start to map-end in the reference was set to maximum 80 bases. For spanning reads, a minimum number of bases at any side of the breakpoint was required to eliminate false positives; >2 for the *GTF2I-BRAF* and *KIAA1549-BRAF* fusions, and >5 for the *DENND2A-GTF2IRD1* fusion. The calculation of relative expression was based on the total number of supporting reads normalized to the number of total raw data reads in each sample (S4 Table).

### RT-PCR and Sanger sequencing

RT-PCR was carried out in 10 μl reactions with 20 ng cDNA and 10 μM of each primer (S1 Table) amplified with AmpliTaqGold Master Mix (Applied Biosystems) by Touch down 65–55 ^0^C PCR using the following cycling conditions: 96°C for 10 minutes; 20 cycles: 94°C for 15 seconds, 65°C (reduced by 0,5°C per cycle from 65°C to 55°C) for 30 seconds, and 72°C for 30 seconds; 25 cycles: 94°C for 15 seconds, 55°C for 30 seconds, and 72°C for 30 seconds. PCR products were separated by electrophoresis on 2% agarose gel containing Gel Red, and photographed. RT-PCR products were sent to GATC Biotech for purification and Sanger sequencing using forward and reverse PCR primers, respectively (www.gatc-biotech.com).

### Fluorescence in situ hybridization

Formalin fixed paraffin embedded tissue (FFPE) sections (2–5 μm) from all six PA cases, were used for interphase FISH analysis. Paraffin sections were pretreated in line with procedures recommended by Abbott, Vysis (Vysis Inc., Downers Grove IL), hybridized with a dual color *BRAF* Break Apart Probe (7q34) (Empire Genomics, Buffalo, NY), counterstained with 4´, 6´, -diamidino-2´-phenylindole dihydrochloride (DAPI), and photographed using a Zeiss Axioplan 2 Imaging fluorescence microscope. Two hundred interphase nuclei were counted by two independent reviewers. The interpretation of intact (normal), and split signals (fusion) was based on accepted international guidelines [24].

### Transient transfection and Western blot

Human embryonic kidney cells (HEK293) were cultured in high glucose DMEM (Thermo Fisher Scientific) supplemented with 10% HyClone bovine growth serum (Thermo Fisher Scientific) at 37°C in 5% humidified CO_2_. Prior to transfection, 4x10^5^ of HEK293 cells were seeded in six well plates. Transient transfection of vector constructs pCMV6-Myc-DDK, pCMV6-BRAF-Myc-DDK (BRAF^WT^) (OriGene Technologies, Rockville, MD, USA), and pCMV6-GTF2I-BRAF-Myc-DDK (synthesized, subcloned and sequenced by Invitrogen GeneArt, Thermo Fisher Scientific) was performed using 2.5μg of each construct, 7.5μl Lipofectamine 3000, and 5μl P3000 reagentfollowing the manufacturer’s protocol (Thermo Fisher Scientific). After 72 hours cells were harvested and lysed using RIPA-buffer supplemented with phosphatase- and protease- inhibitors (Thermo Fisher Scientific). Protein lysates (30 μg) were resolved on 4–20% precast gels (Bio-Rad Laboratories) and transferred onto 0.45 μm PVDF membranes (Thermo Fisher Scientific). Western blot was performed using antibodies with ECL-detection (Supersignal West Maximum Fempto, Thermo Fisher Scientific) as follows: FLAG-DDK M2 mouse mAb (1:750, #F3165, Sigma Aldrich), GAPDH rabbit Ab (1:500, #sc-25778, Santa Cruz Biotechnology), phosphorylated-p44/42 MAPK (ERK1/2) (Thr202/Tyr204) rabbit mAb (1:500, #4370), p44/42 ERK (ERK1/2) rabbit mAb (1:1000, #4695) from Cell Signaling Technology, Amersham HRP-conjugated mouse or rabbit IgG (1:10 000, #NA931/NA934), GE Healthcare Life Sciences). Chemiluminescent signal from membranes were imaged using a LAS-400 imaging system (Fujifilm). Western blot was performed in triplicates for each sample and quantified using Image Studio Lite v 5.2.5 (www.licor.com/bio/products/software/image_studio_lite/). The pERK levels were calculated relative to the total ERK (tERK) protein expression, and normalized against GAPDH as loading control.

### Immunohistochemistry

For routine pathological examination and assessment of tumor cell content, hematoxylin and eosin staining (HE) was performed. FFPE sections (5 μm) from all six cases were deparaffinized, rehydrated and antigen retrieved with citrate-based solution (low pH) (Vector Laboratories, Burlingame, CA, USA). Endogenous peroxidase activity was blocked with ready-to-use EnVision hydrogen peroxide (Dako) followed by incubation at 4°C overnight, with primary antibody against phosphorylated-44/42 MAPK (ERK1/2) (Thr202/Tyr204) rabbit mAb (1:400, #4370, Cell Signaling Technology, Danvers, MA). The antibody-antigen complex was visualized using the ready- to- use Dako EnVision FLEX HRP labeled polymer system and chromogen diaminobenzidine (DAB) staining, according to the manufacturer’s protocol (Dako, Glosterup, Denmark). Normal human brain cerebellum tissue sections from autopsy specimen were included as a reference tissue (NBP2-42613, Novus Biologicals, a Biotechne brand, www.novusbio.com).

## Results

### Characterization of PA cases and *BRAF* status

**Fig 1:**
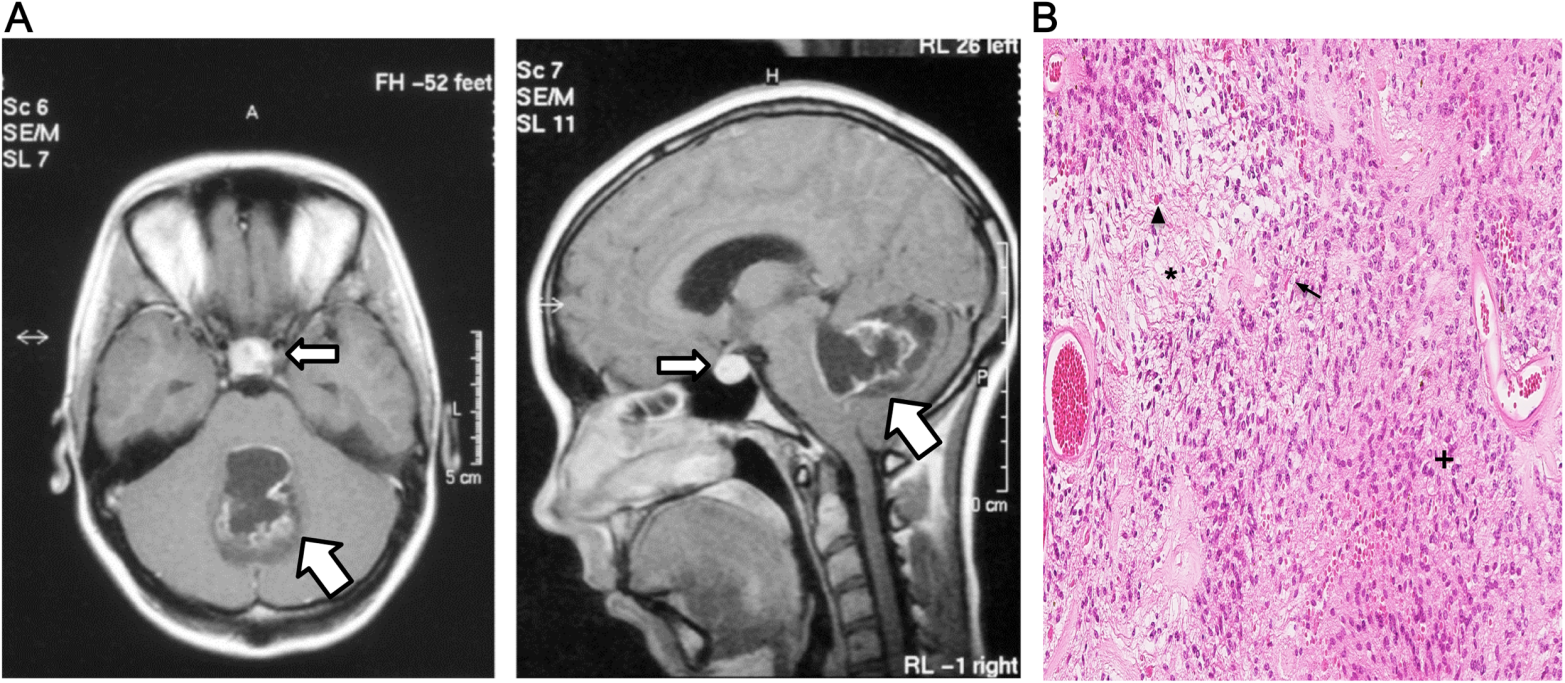
(A) Axial (left) and sagittal (right panel) MR images of PA3 tumor (T1-weighted, contrast enhanced) showing a cerebellar tumor (fat arrow), 4,5 x 3 x 3 cm, partly cystic with irregular contrast enhancement. In sella turcica, a 1.5 cm in diameter intensely contrast enhancing round tumor with a 0.5 cm cystic component is seen (slim arrow). The S-prolactin level was elevated, 1510 mIU/l (ref < 400), indicative of a pituitary prolactinoma. (B) Hematoxylin and Eosin staining of case PA3, demonstrates biphasic pattern with compact (+) and loose (*) areas, including Rosenthal fibers (arrow) and eosinophilic granular bodies (arrow head).

The Magnetic Resonance (MR) images showed classical features of PA in the cerebellum, and diagnosis was confirmed with histhopathological analysis; biphasic tumor areas with compact and loosen areas, Rosenthal fibers and eosinopfilic granular bodies (Fig 1, Table 1, and S1 Fig). Tumor cell content was estimated to be around 70% on average (>90% in tumor areas) in all six PA cases, based on Hematoxylin-Eosin-stained FFPE sections (S1 Fig). Each sample was investigated with RT-qPCR for presence of the three most common *KIAA1549-BRAF* mRNA fusion junctions (16–9, 15–9, and 16–11) [25] and *BRAF*V600E point mutation status (Table 2). The *KIAA1549-BRAF* 16–9 fusion was detected in five out of six cases (PA 1–2, PA 4–6). None of the samples was positive for *BRAF*V600E. Hence, a causative *BRAF* alteration could be identified in five out of six cases, whereas in case PA3 the genetic background was unknown. The cerebellar tumor in PA3 was partly cystic with irregular contrast enhancement. In addition to the PA tumor, a pituitary prolactinoma was revealed in the sella turcica, supported by the elevated S-prolactin level in the patient (Fig 1A). Co-occurrence of two or more brain tumors with different histological features is rare, although a few cases have been reported [26]. Moreover, in patients harboring pituitary tumors co-prevalence of other primary tumors is demonstrated to be significantly higher than expected in the general population [27]. Unfortunately, no material was available from the prolactinoma and hence only the cerebellar tumor (PA) from case PA3 was studied in the present paper. Patients underwent gross total resection of the Astrocytoma in the cerebellum, and all patients are alive with no evidence of disease. More information about event and other treatment can be found in Table 1.

**Table 2:** Summary of results from five different methods for BRAF alteraration detection.

	castPCR	qPCR			RNA-seq					CNV (SNP array)		FISH	
Sample ID	BRAF mut	KIAA1549-BRAF fusion			Number of fusions from FusionCatcher			Detection of BRAF fusion		Rearrangements		BRAF split probe	
	V600E	Ex 16–9	Ex 15–9	Ex 16–11	Pred fusions Prel. List	Pred fusions Final list	Coding fusions Final list	Prel. list	Final list	Gain/Loss	Breakpoints	Detection of rear.	% cells with rear.
**PA1**	Neg	Pos	Neg	Neg	8159	17	1	**Y**	N	1 gain (7q34)	KIAA1549-BRAF	**Y**	67%
**PA2**	Neg	Pos	Neg	Neg	8173	24	0	**Y**	N	1 gain (7q34)	KIAA1549-BRAF	**Y**	76%
**PA3**	Neg	Neg	Neg	Neg	11527	34	2	N	N	2 gains (7q11.23 & 7q34)	GTF2I-BRAF & DENND2A-GTF2IRD1	**Y**	45%
**PA4**	Neg	Pos	Neg	Neg	4226	17	0	N	N	1 gain (7q34)	KIAA1549-BRAF	**Y**	55%
**PA5**	Neg	Pos	Neg	Neg	9360	29	1	**Y**	N	1 gain (7q34)	KIAA1549-BRAF	**Y**	55%
**PA6**	Neg	Pos	Neg	Neg	12726	47	0	**Y**	N	Neg	Neg	**Y**	40%

Detection of BRAF-alterations with four methods: Competitive Allele-Specific TaqMan PCR (castPCR), quantitative PCR (qPCR), RNA sequencing (RNA-seq), Copy number variation (CNV) analysis with Single nucleotide polymorphism array (SNP-array), Fluorescense in situ hybridization (FISH). BRAF mut: BRAF point mutation; V600E = amino acid exchange; Neg = negative; Pos = Positive; Ex = Exon junction; Pred fusions = Number of Predicted fusions in Preliminary and Final list from FusionCatcher; Coding fusions: Fusions that will result in a new protein-coding fusion transcript; Detection of BRAF fusion = Presence of KIAA1549-BRAF or GTF2I-BRAF fusion in lists; Y = yes; N = No; Gain/Loss according to ChAS browser segment filter settings; BRAF split probe = BRAF break apart FISH result; Detection of rear. = Detection of rearrangement, Y = yes, n.d. = not determined; % of cells with rear. = frequency of rearrangement ("split probe"-positive cells when calculating 200 random interphase nuclei in the microscope, see methods for details).

### Fusion detection by CNV and RNA-seq

**Fig 2:**
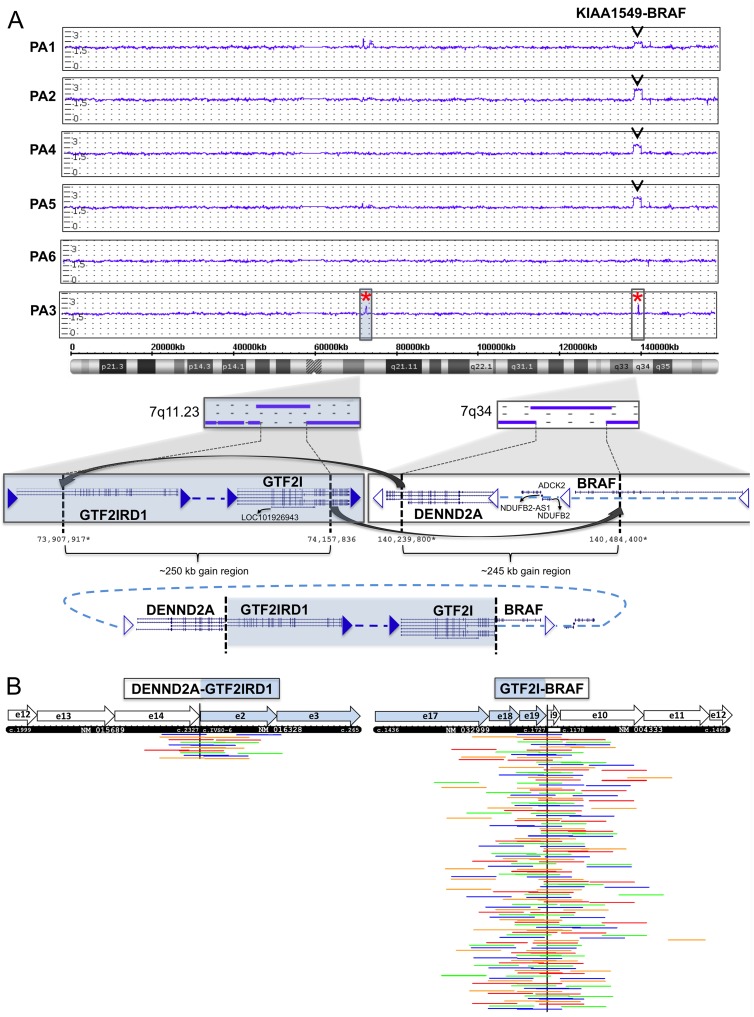
**(**A**)** Copy number variation (CNV) genomic profiling with CytoScan HD SNP arrays. The weighted log2 ratio, smooth signal, and allele difference plot for chromosome 7 is shown for all six PA samples. Four out of five samples show the *KIAA1549-BRAF* duplication in 7q34. In case PA3 two novel duplications of approximately 250kb each were detected; one in 7q11.23 with breakpoint within *GTF2IRD1* and *GTF2I*, and one in 7q34 with breakpoint within *DENND2A* and *BRAF*. The two duplicated regions give rise to two fusion junctions; *DENND2A-GTF2IRD1* exons 14–2 and *GTF2I-BRAF* exon 19–10, probably through a circularization event followed by incorporation into the genome. The breakpoint positions are according GRCh37/Hg19 at UCSC Genome Browser (https://genome.ucsc.edu). Positions marked with star (*) are approximate by manual inspection in the ChAS software. (B**)** Supporting reads for the *DENND2A-GTF2IRD1* 14–2 and *GTF2I-BRAF* 19–10 fusion junctions in RNA sequencing data from case PA3. Spanning and split read pairs supporting the junction were extracted by BLAT and were aligned to 600bp of the predicted mRNA/cDNA sequence for each fusion. A schematic presentation of the mRNA junction is presented by the black box, showing exons (e), positions in cDNA, and GenBank accession numbers (*DENND2A*: NM_015689, *GTF2IRD1*: NM_016328, *GTF2I*: NM_032999), *BRAF*: NM_004333). Each row represent read pairs (or single reads) supporting a unique template. The RNA-seq data contained a total of 10 unique supporting read pairs/reads for *DENND2A-GTF2IRD1* gene fusion and a total of 109 unique supporting read pairs/reads for the *GTF2I-BRAF* gene fusion. Only reads supporting the fusions are shown. Read pairs are in the same color. e = exon; i = intron.

CNV screening with high-resolution SNP arrays caught the previously detected *KIAA1549-BRAF* 16–9 fusion in four out of five cases. One sample (PA6) showed a flat profile (no gains or losses on chromosome 7) (Fig 2A). Applying the FusionCatcher tool to RNA-seq data, none of the *KIAA1549-BRAF* 16–9 fusions could be identified in the final lists (Table 2 and S2 Table).

Screening for novel fusions in the *KIAA1549-BRAF*-negative PA3 case was performed by a combined CNV and RNA-seq data analysis (see Material and Methods for details). RNA sequencing analysis with FusionCatcher predicted two coding fusions; *DENND2A-GTF2IRD1* and *GFAP-SPARC*. Analyzing SNP array data of PA3, two CNV duplications of ∼250kb and ∼245kb each were identified; one involving *GTF2I* and *GTF2IRD1* in 7q11.23 and a second involving *DENND2A* and *BRAF* in 7q34 (Fig 2A and Table 2). These CNV duplications verified the novel *DENND2A-GTF2IRD1* 14–2 fusion junction detected by RNA-seq, but also indicated a *BRAF* fusion formed by the same rearrangement event; *BRAF-GTF2I*. According to the CNV profile the two duplications were probably linked together into one fragment by a circularization event and somehow incorporated back into the genome (Fig 2A). No CNV gains or losses could be detected around the *GFAP-SPARC* fusion (17q21.31, 5q33.1) proposed by FusionCatcher, and since the junction could not be verified by RT-PCR this predicted fusion was excluded for further analysis.

In order to identify the precise fusion junction between *GTF2I* and *BRAF*, a 30 bp sequence from the 5’ junction site in *BRAF* (exon 10) was used to search for and extract all matching spanning reads present in the RNA-seq data (se Material and Methods for details). BLATing the extracted sequences revealed a novel gene junction between exon 19 in *GTF2I* and exon 10 in *BRAF*, supported by 109 reads or read pairs matching both *GTF2I* and *BRAF* (Fig 2B). The fusion also included a small 17 bp segment from *BRAF* intron 9, producing an *in-frame* fusion.

### Confirmation of the novel fusion junctions

**Fig 3:**
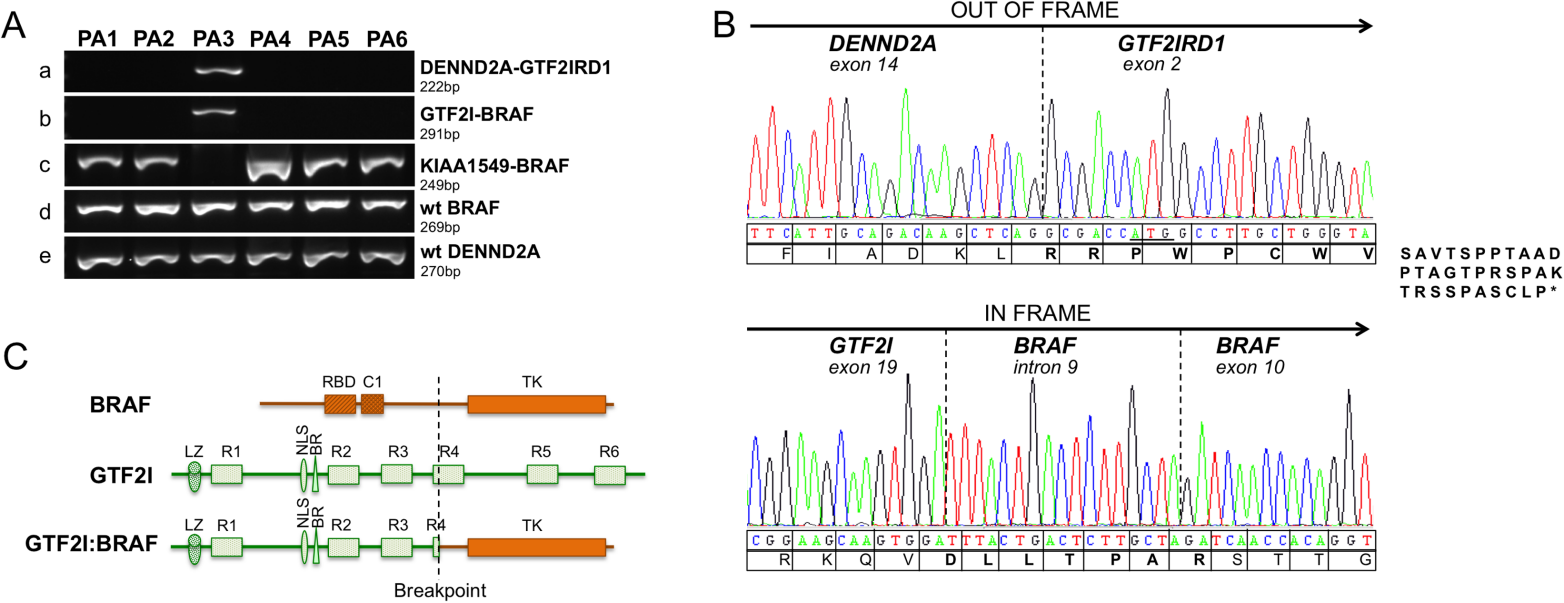
**(**A**)** RT-PCR verification of gene fusions in six PA cases. Gene fusion PCR products of a) *DENND2A-GTF2IRD1* 14–2 (222 bp), b) *GTF2I-BRAF* 19–10 (291 bp), c) *KIAA1549-BRAF* 16–9 (249 bp), and wild type (wt) positive control products of d) *BRAF* and e) *DENND2A*. e = exon; i = intron. (B**)** Sanger sequencing of RT-PCR fusion junction products. Translation of codons is shown below the electropherograms. Upper panel: The *DENND2A-GTF2IRD1* 14–2 junction result in an *out-of-frame* truncated protein generating 41 new amino acids of the C-terminal of DENND2A. The ATG translation starting site in *GTF2IRD1* is underlined. Lower panel: The *GTF2I-BRAF* 19–10 *in-frame* junction results in a putative protein involving an integrated sequence of 16bp from *BRAF* intron 9. New amino acids produced by the junctions are marked in bold. (C**)** Schematic illustration of the GTF2I and BRAF protein domains and localization of the fusion breakpoints. The TFII-I GTF2I protein (998 amino acids (aa), NP_127492) consists of six helix-loop-helix–like domains (R1-R6); the DNA binding domain basic region (BR); the nuclear localization signal (NLS); the leucine zipper domain (LZ). The BRAF (766 aa, NP_004324) consists of the Ras binding domain (RBD), the Phorbol ester/diacylglycerol binding zink finger domain (C1) and the tyrosine kinase (TK) domain. The breakpoint locus in GTF2I (position 575 aa) and BRAF (position 394 aa) is marked by a dashed vertical line. The GTF2I-BRAF putative fusion protein is 955 aa long and contains the TFII-I DNA binding domains (R1-R3, BR and LZ) and nuclear localization (NLS) from GTF2I joined to the tyrosine kinase (TK) domain of BRAF. Domains and positions are according to NextProt (http://www.nextprot.org, accessing date 2016-04-25).

All fusion transcripts were verified by RT-PCR. Case PA3 was positive for PCR products from *GTF2I-BRAF* 19–10 (291bp) and *DENND2A-GTF2IRD1* 14–2 (222bp; Fig 3A). In accordance to results from qPCR, the PCR product of *KIAA1549-BRAF* 16–9 (249bp) could be detected in the five remaining PA cases. To verify and confirm the identity of the two novel fusion junctions, each transcript was sequenced by Sanger (Fig 3B). The *DENND2A-GTF2IRD1* 14–2 fusion, in which *DENND2A* is joined to six base pairs upstream of the ATG translation start in *GTF2IRD1*, produces an *out-of-frame* reading sequence with 41 new amino acids in the C-terminal of DENND2A. The truncated putative 815 aa DENND2A protein is similar in length as the DENND2A transcript version 3 (795 aa, NM_001318053). The *GTF2I-BRAF* 19–10 junction produces an *in-frame* reading sequence involving an inclusion of a 17 bp segment from *BRAF* intron 9. The putative GTF2I-BRAF fusion protein is 955 aa in length, containing the TFII-I DNA binding domains (R1-R3, BR and LZ) and nuclear localization (NLS) from GTF2I joined to the tyrosine kinase (TK) domain of BRAF (Fig 3C).

### Expression of fusions

**Fig 4:**
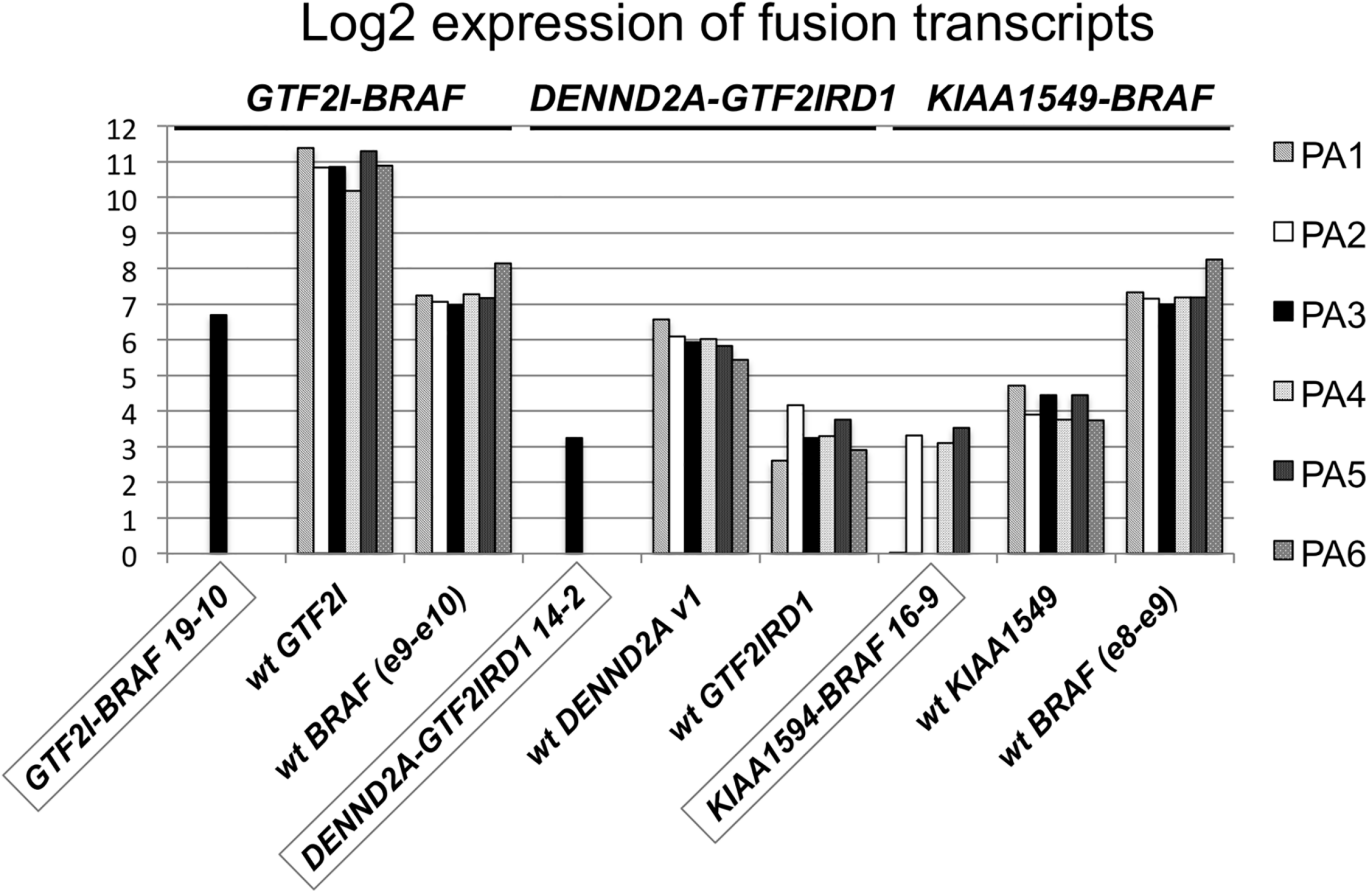
Expression analysis of fusion transcripts based on RNA-seq data. Log2 RNA-seq expression data for three fusion transcripts; *GTF2I-BRAF* 19–10, *DENND2A-GTF2IRD1* 14–2, *KIAA1549-BRAF* 16–9 compared to wild type fusion partner genes in six PA cases. Expression data was calculated as total number of supporting reads normalized to the total number of raw reads in each sample. Exon-exon junction in genes are as follows; *GTF2I-BRAF* 19–10 (exon 19- exon 10), *GTF2I* (exon 19- exon 20), *BRAF* e9-e10 (exon 9- exon 10), *DENND2A-GTF2IRD1* 14–2 (exon 14- exon 2), *KIAA1549-BRAF* 16–9 (exon 16- exon 9), *DENND2A* v1 (transcript version 1, exon 14- exon 15), *KIAA1549* (exon 16- exon 17), *BRAF* e8-e9 (exon 8- exon 9).

All sequence reads around fusion break points, both “spanning” reads and “split” read pairs, were extracted from the RNA-seq data. Expression of each fusion gene and corresponding wild type (wt) genes was calculated from the number of supporting reads, and normalized to the number of raw data reads (Fig 4 and S4 Table). In case PA3, 109 fragments supported the *GTF2I-BRAF* fusion (115 spanning reads, 9 split read pairs) and 11 reads supported the *DENND2A-GTF2IRD1* fusion (11 spanning, 0 split read pairs) (Fig 2B and S4 Table). The three fusions showed a lower expression of fusion transcript than the 3’ wild type partner; *GTF2I-BRAF* versus *GTF2I* (18-fold), *DENND2A-GTF2IRD1* versus *DENND2A* ver.1 (6-fold), *KIAA1549-BRAF* versus *KIAA1549* (2-fold). The expression of the *GTF2I-BRAF* fusion (in PA3) indicates an elevated expression compared to *KIAA1549-BRAF* (13-fold, cases PA 1–2 and PA 4–5; Fig 4). Overall, the *KIAA1549-BRAF* expression was fairly low in all four positive cases with a maximum of 11 supporting reads in case PA5. Comparing the expression of *GTF2I* to *KIAA1549* in all cases showed a 106-fold higher expression of *GTF2I*, indicating a stronger promoter (Fig 4). Although the PA6 case was positive for the *KIAA1549-BRAF* 16–9 fusion by qPCR (Table 2) and RT-PCR (Fig 3A), no supportive reads could be found in the RNA-seq data and this case also displayed a flat SNP-array profile (S4 Table and Fig 2A). An additional independent RT-PCR of sample PA6 was performed, and Sanger sequencing the PCR product confirmed the presence of the *KIAA1549-BRAF* 16–9 fusion (S2 Fig). Notably, the wild type *BRAF* expression was elevated by 2-fold in the PA6 case compared to the other five cases (Fig 4).

### *BRAF* break apart FISH analysis

**Fig 5:**
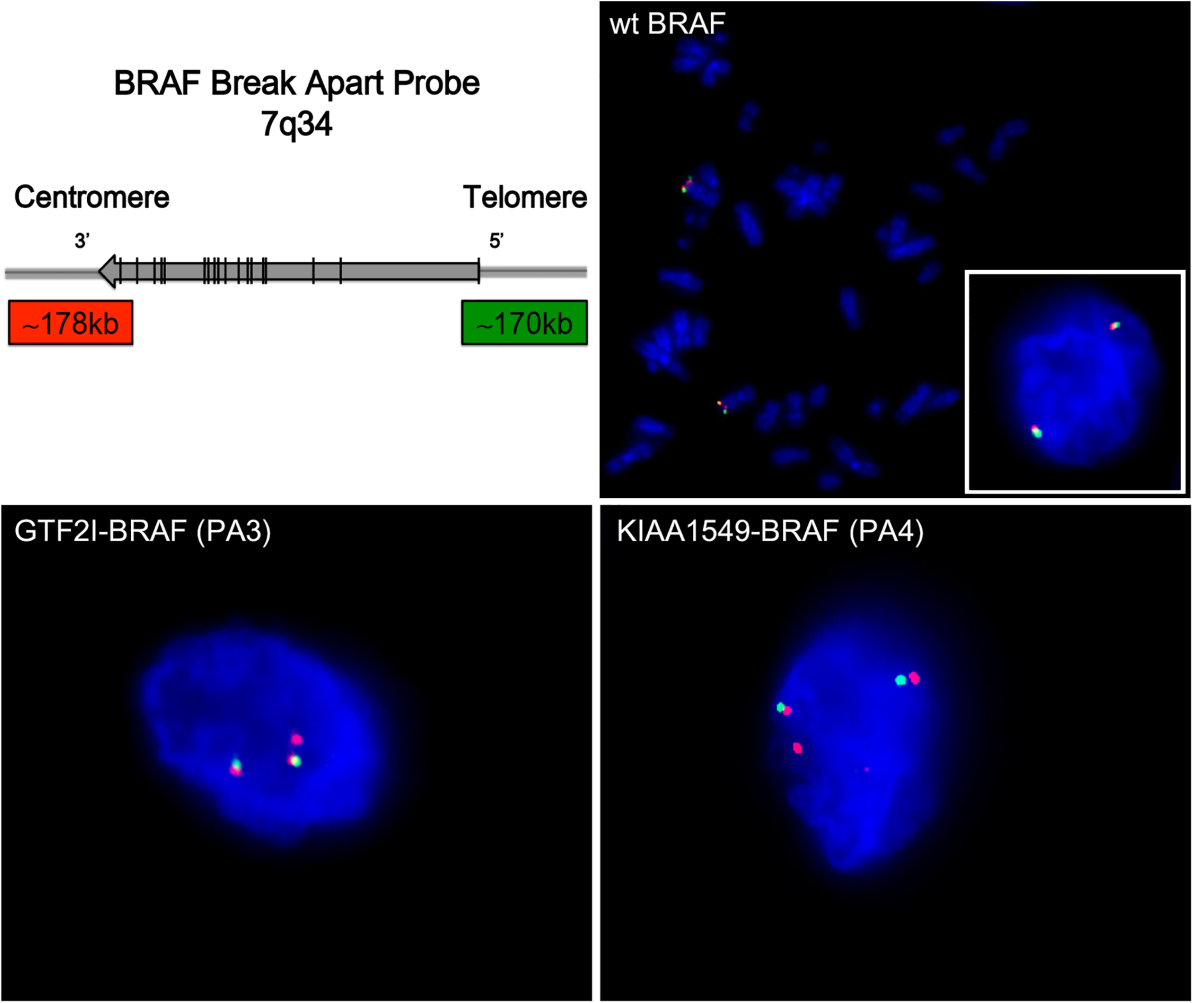
FISH analysis of *BRAF* fusions with *BRAF* break apart assay. Upper left: Schematic presentation of the *BRAF* break apart assay, consisting of a 5’ 170 kb green probe and a 178 kb 3’ red probe in 7q34. Upper right (wt BRAF): Metaphase FISH of normal control and Interphase FISH of fusion-negative cell (right corner) showing two wild type *BRAF* alleles, displayed as a merged (yellow) or two adjacent green (5’)/red (3’) signals. Lower panels: Fusion-positive tumor cells (*GTF2I-BRAF* in PA3 and *KIAA1549-BRAF* in PA4) showing the *BRAF* split pattern; two normal *BRAF* alleles green /red signals, as well as one additional split *BRAF* red signal representing the duplicated 3’ region in the fusion gene. The same split signal pattern is seen for different types of *BRAF* fusions; *GTF2I-BRAF* in case PA3 and *KIAA1549-BRAF* in cases PA1-2 and PA4-6 (S3 Fig). Tissue sections were counterstained with DAPI (blue).

Out of the two novel fusions, the *in-frame GTF2I-BRAF* fusion was considered as the most plausible explanation factor for tumorigenesis in case PA3. Therefore, the new *GTF2I-BRAF* rearrangement together with the known *KIAA1549-BRAF* (16–9) fusion was further validated using *BRAF* Break Apart FISH assay on PA cases (Fig 5 and S3 Fig). First, the *BRAF* Break apart probes were verified to be located in 7q34 by metaphase FISH on normal blood cells (Fig 5). Normal and fusion-negative nuclei displayed two pairs of merged (yellow) or adjacent signals green/red (5’/3’), representing the two wild type *BRAF* alleles. Fusion-positive nuclei in tumor sections display the *BRAF* break apart pattern; two pairs of merged (yellow) or adjacent signals green/red (representing 5’/3’wt *BRAF* alleles), and one additional split red (3’) signal indicating a duplicated copy of the 3’ *BRAF* region. All six PA cases showed the break apart pattern, although the split 3’ signal could be close (*e*.*g*. PA2, PA5) or more distant (*e*.*g*. PA4) away from the normal allele in 7q34, probably depending on the integration site of the duplicated region (S3 Fig). Around 50% of cells in all PA cases were fusion-positive, *i*.*e*. showing split signal of 3’ *BRAF* (Table 2).

### Activation of MAPK pathway

**Fig 6:**
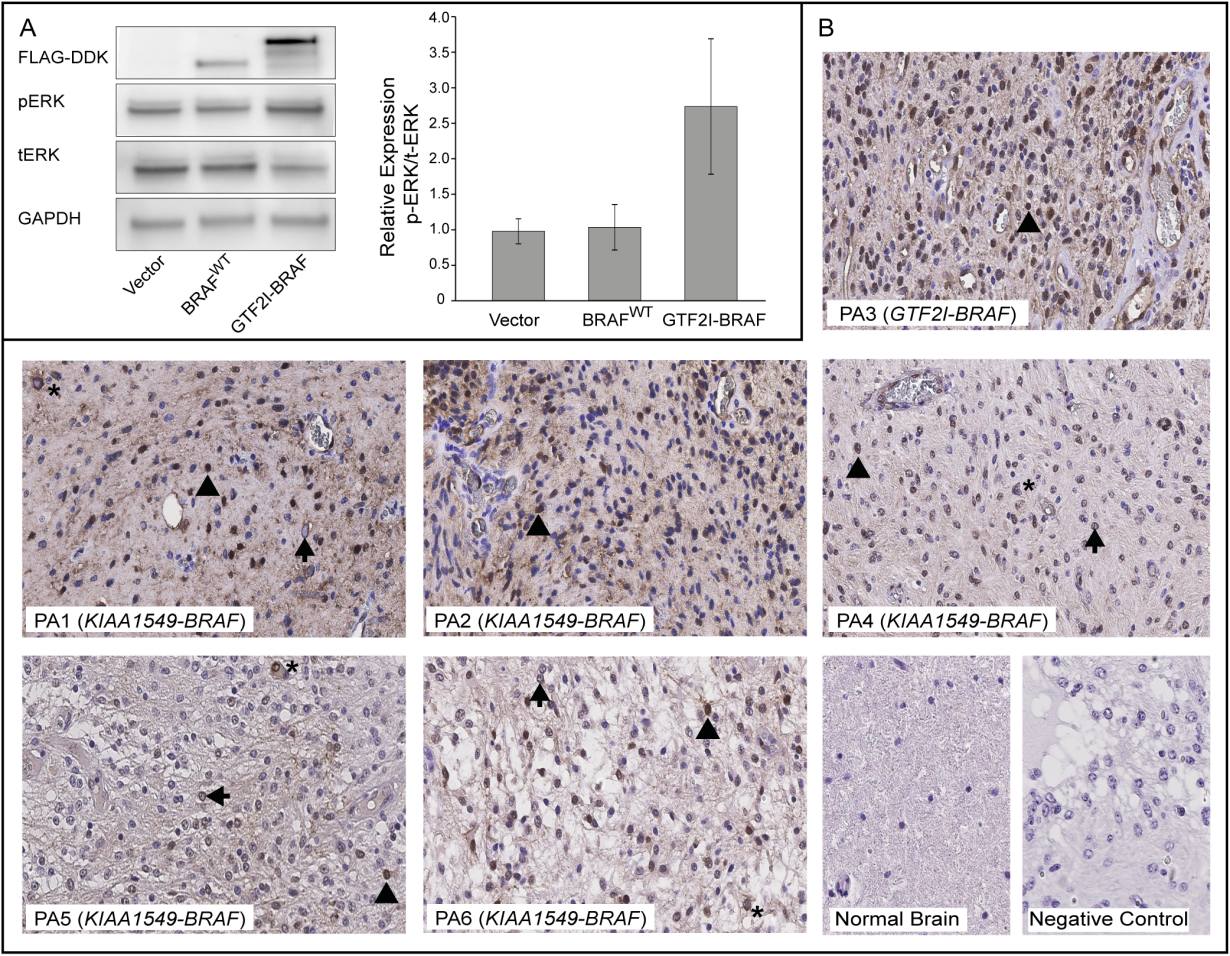
Activation of the MAPK pathway. **(**A**)** Western Blot of protein lysates from HEK293 cells transiently transfected with pCMV6-Myc-DDK (empty vector), pCMV6-BRAF-Myc-DDK (BRAF^WT^) or pCMV6-GTF2I-BRAF-Myc-DDK (GTF2I-BRAF) were probed with antibodies against FLAG-DDK, phosphorylated ERK-Thr202/Tyr204 (pERK), total ERK (tERK) and GAPDH. Bars show relative mean pERK/tERK protein expression for each construct performed in triplicates (mean±SEM) after normalization to GAPDH. (B**)** Activation of the MAPK pathway in PA tumor tissue. FFPE sections from the six primary PA cases were immunostained with phosphorylated-ERK-Thr202/Tyr204 (pERK) antibody. Tumor tissue (PA1-6) showing perinuclear (arrow), nuclear (arrow head) and to lesser extent cytoplasmic (*) pERK staining. Normal human brain cerebellum reference tissue section from an autopsy specimen showing negative staining for pERK. Negative control with omitted primary antibody showing negative staining for pERK. Some endothelial cells were also positive for pERK. Original magnification x400.

To elucidate expression and the MAPK activating potential of the *GTF2I-BRAF* fusion protein, HEK293 cells were transiently transfected with GTF2I-BRAF, BRAF^WT^, and empty vector constructs (pCMV6-Myc-DDK) respectively, and the level of pERK was measured with Western blot. Using anti- FLAG antibody the GTF2I-BRAF protein showed a band shift at the size of ~120 kDa, while the BRAF^WT^ protein was detected at ~95 kDa. Cells expressing the GTF2I-BRAF fusion protein showed an elevated expression of pERK/tERK in comparison to cells transfected with BRAF^WT^ and empty vector (Fig 6A). In addition, MAPK pathway activation was investigated in primary PA tissue sections with Immunohistochemistry. The pERK staining was increased in all six PA tumors compared to normal brain cerebellum regardless of the BRAF fusion type. pERK expression was predominantly found to be perinuclear and nuclear and to lesser extent cytoplasmic in tumor cells. Some blood vessels were also positive for pERK expression (Fig 6B). When omitting the primary antibody no staining was observed.

## Discussion

MAPK signaling pathway is the most important pathway for regulating cell growth, proliferation, apoptosis, and differentiation [28]. BRAF, a downstream target of RAS proteins, is a common target for activating mutations and fusions in diverse cancer forms including PA [29, 30]. The most frequent event in tumorigenesis of PA is *BRAF* gene rearrangements formed by duplication or deletions on chromosomal region 7q34, and *KIAA1549-BRAF* 16–9 is found in the majority (∼60%) of PA cases [7, 9–11]. In the present study we report for the first time a *GTF2I-BRAF* fusion in a pediatric brain tumor. The fusion was formed by rearrangement of two duplication events on 7q; one in 7q11.23 and one in 7q34. Partner genes in 7q34 (*e*.*g*. *BRAF*, *KIAA1549*) are transcribed in the telomere to centromere direction, and linear duplications/translocations may simply form fusions. In contrast, *GTF2I* (general transcription factor 2I) and neighboring genes in 7q11.23 are transcribed in opposite direction requiring an inversion in addition to the duplication and translocation to form a functional gene. The *GTF2I* intron 19 where the breakpoint occurred is also small compared to the common breakpoint introns of *KIAA1549*. These differences between *KIAA1549* and *GTF2I* may explain why the former partner gene is much more common.

The new *GTF2I-BRAF* 19–10 fusion generates a putative protein containing the TFII-I DNA binding domains from GTF2I joined to the tyrosine kinase (TK) domain of BRAF. Interestingly, another *GTF2I-BRAF* fusion (exon 4–10) has been reported in one primary melanoma case [29], supporting a role of this fusion partnership in tumor development. Similar to other BRAF/RAF1 fusions in PA, the GTF2I-BRAF fusion protein lacks N-terminal inhibitory domain leading to constitutive active BRAF kinase and increased MEK/ERK signaling [7, 10, 12, 16]. In line with defined functional properties of BRAF fusions, the current study demonstrates GTF2I-BRAF 19-10-expressing HEK293 cells to exhibit increased pERK levels, thus confirming a role of GTF2I-BRAF in constitutive activation of the MAPK pathway. In addition, Immunohistochemistry show that the PA cases, harboring either *KIAA1549-BRAF* or *GTF2I-BRAF*, displays an elevated expression of pERK compared to normal brain tissue, further supporting an increase in MAP kinase activity mediated by *BRAF* fusions. Notably, the pERK was mainly located in the nucleus, which is essential for activation of transcription targets [31].

*BRAF* gene fusions are reported to involve many different 5’ partners, (*e*.*g*. *KIAA1549*, *FAM131B*, *RNF130*, *CLCN6*, *MKRN1*, *GNAI1*) although *KIAA1549* is the most prevalent one [7–9, 12–15]. The possible role of BRAF fusions partners in PA tumorigenicity is largely unknown. Shin et al. [32] showed that the BRAF kinase domain alone, without fusion partner, was not able to induce tumors in mice indicating a certain role of fusion partners. The new GTF2I fusion partner has been implicated in regulation of several cellular processes involving growth factor induced signal transduction, proliferation, apoptosis, angiogenesis, TGFB signaling, and immune response [33]. Interestingly, GTF2I has also been shown to regulate nuclear translocation of ERK1/2 upon mitogenic signaling [31], and indicates an indirect role of GTF2I in activation of the MAPK pathway. Occurrence of additional *GTF2I*-fusions; *GTF2I-BRAF 4–10* (primary melanoma) [29], *GTF2I-NCOA2* (soft tissue angiofibroma development) [34] and *GTF2I-RARA* (acute promyelocytic leukemia) [35], further supports a oncogenic role. In the present study, the *GTF2I* promoter appears to be stronger than the *KIAA1549* indicated by the elevated expression of both the *GTF2I-BRAF* fusion and the *GTF2I* transcript. However, no an altered disease progression could be observed in the index case (PA3) harboring the *GTF2I-BRAF* fusion. The patient underwent radical surgery of the Astrocytoma in the cerebellum in November 2003, and has not relapsed since then.

The two CNV gains on 7q also resulted in an additional rearrangement in the same tumor case; *DENND2A-GTF2IRD1* 14–2. *DENND2A* (DENN/MEDD domain 2A), located next to *BRAF*, encodes a guanine nucleotide exchange factor (GEF) that activates members of the Rab pathway. The small Rab GTPases regulate growth factor signaling and cell mobility through intracellular vesicle transport, and has important roles in migration and invasion of tumor cells [36]. Since the *DENND2A-GTF2IRD1* putative fusion product will result in a truncated DENND2A protein, the consequence of this gene fusion is probably moderate and is not expected to play a role in PA tumorigenesis. Yet, inclusion of *DENND2A* in a 7q-rearrangement in PA has previously been reported by Cin and colleagues [10].

In recent years, RNA sequencing has become a prevalent method for fusion gene detection in cancer [37]. Indeed, transcriptomic sequencing has taken the fusion gene discovery into another level since it allows for detection of all (balanced or unbalanced) expressed rearrangements, including alternative splice variants resulting from a fusion event [37]. However, one main limitation of RNA sequencing is that it cannot detect rearrangement events involving non-transcribed regions. Also, since the dynamic range of expression is broad and tissue-specific, fusion genes expressed at low level can be difficult to detect. Current available bioinformatics algorithms for fusion discovery often report a very high number of false positive chimeras [38], or may be to stringent to detect the fusions that are actually present in the data [15]. This stresses the importance of evaluation and fine-tuning of bioinformatic pipeline for fusion gene discovery [39]. The new *GTF2I-BRAF* 19–10 fusion identified in present study would probably not have been discovered by RNA sequencing and CNV analysis alone. One issue that complicated the finding of this fusion gene with RNA-seq was the occurrence of pseudogenes for *GTF2I* on different chromosomal location, and it was excluded by the FusionCatcher algorithm. Moreover, the detection of *GTF2I-BRAF* fusion with CNV analysis was also problematic since the duplications generating the fusion were quite small, hence required modification of settings and thorough manual inspection. On the hand, the larger duplication producing the most prevalent *KIAA1549-BRAF* fusion was clearly detected by the SNP-arrays in most cases, but could not be captured by the FusionCatcher algorithm due to the low expression levels in the RNA-seq data. Our results are in line with Lin et al. [14] who demonstrated that *KIAA1549-BRAF* fusions is expressed at lower levels than *BRAF*, but at only slightly lower levels than the *KIAA1549* promoter. In summary, occurrence of pseodogenes and low expression/few supporting reads [14, 15] complicates fusion detection by the RNA sequencing method.

Due to the high prevalence of *BRAF* rearrangements in pediatric PA and the documented better clinical outcome of *KIAA1549-BRAF* positive cases, the *BRAF* fusions have been suggested as a prognostic marker and supplement tool for better diagnostics [4, 17, 19, 20]. This emphasizes the need for reliable detection of variant *BRAF* rearrangements in clinical routine irrespectively of its fusion partner or exon-exon junction. The *BRAF* break apart FISH probe used in this paper to validate presence of *BRAF* fusions was able to detect both *KIAA1549-BRAF* and *GTF2I-BRAF* with the same sensitivity. The advantage of FISH method compared to qPCR, CNV analysis and RNA sequencing is that this method is a robust and informative diagnostic tool to indicate *BRAF* rearrangements independent of the fusion partner, fusion junction, size of duplication, or expression of the fusion gene. Moreover, *BRAF* break apart FISH probe method renders the possibility to assess the number of fusion-positive cells and is applicable for FFPE sections available in clinical routine. Disadvantages are that neither the fusion partner nor the fusion consequence (*in-frame* or *out-of-frame*) is detected. But complementary qPCR can be used to detect the most common *KIAA1549-BRAF* fusions in PA [21]. Nevertheless, the sensitivity and specificity of the break apart FISH method as a routine method needs further validation in larger PA cohorts.

The high occurrence and impact of *BRAF* rearrangements in pediatric gliomas, essentially PA, not only open the possibility for better diagnostics but also provides an opportunity for targeted therapy. Selective B-Raf inhibitors such as Vemurafinib and Sorafenib that have been developed for the deregulated B-Raf malignancy in other tumor types (*e*.*g*. melanoma), can be used for management of approximately 8% of all PA harboring *BRAF*V600E mutation [14, 40]. However, Raf inhibitors have been reported to generate a paradoxal activation of the MAPK pathway in the cells expressing wild type BRAF and BRAF fusions [41, 42]. Due to this fact, Phase II trial with Sorafenib for treatment of recurrent or progressive low-grade astrocytoma’s was discounted [43]. Instead, MAPK/ERK kinase (MEK) inhibitors are currently in clinical trials and can provide alternative therapy independent of BRAF status (ClinicalTrials.gov identifier NCT01386450; NCT01089101).

The knowledge about molecular genetics behind development of PA has increased tremendously in recent years. The new *GTF2I-BRAF* 19–10 fusion reported in this paper further emphasizes the central role of *BRAF* in tumorigenesis, and the MAPK pathway as a promising therapeutic target in PA. Moreover, the occurrence of an increasing number of *BRAF* fusion variants and possibility for MAPK pathway targeted therapy highlights the importance of a robust method for fast and cost-effective detection of *BRAF* deregulations to guide diagnosis, prognosis, and accurate targeted therapy.

## Supporting information

S1 Fig Histopathological Hematoxilyn-Eosin sections of six PA cases. (PDF)

S2 Fig Sanger sequencing of RT-PCR KIAA1549-BRAF fusion product in PA6. (PDF)

S3 Fig *BRAF* fusion detection with *BRAF* Break Apart FISH in six PA cases. (PDF)

S1 Table Primers used for RT-PCR amplification and Sanger sequencing analysis of fusion products. (XLSX)

S2 Table FusionCatcher candidate output lists. (XLSX)

S3 Table BLAT sequences and fusion targets for RNA sequencing data. (XLSX)

S4 Table Expression of fusion transcripts based on RNA-seq data. (XLSX)

## Abbreviations

PA: Pilocytic astrocytoma
MAPK: mitogen activated protein kinase
CNV: copy number variation
FISH: Fluorescence in situ hybridization
RNA-seq: RNA sequencing
LGG: low-grade gliomas
WHO: World Health Organization
NF1: Neurofibromatosis type 1
qPCR: quantitative Reverse Transcriptase RT, -PCR; high-resolution
SNP: Single Nucleotide Polymorphism arrays
CGH: Comparative Genomic Hybridization
SDS: Sequence Detection System; quantitation cycle
castPCR: Competitive Allele-Specific TaqMan PCR
ChAS: Affymetrix Chromosome Analysis Suite
FFPE: formalin fixed paraffin embedded tissue
DAPI: 4´,6´,-diamidino-2´-phenylindole dihydrochloride
DAB: diaminobenzidine
pERK: phosphorylated ERK-Thr202/Tyr204)
NSC: neural stem cells
GEF: guanine nucleotide
TK: tyrosine kinase
