# Correction: A new *GTF2I-BRAF* fusion mediating MAPK pathway activation in pilocytic astrocytoma

**DOI:** 10.1371/journal.pone.0184715

**Published:** 2017-09-07

**Authors:** Tajana Tešan Tomić, Josefin Olausson, Annica Wilzén, Magnus Sabel, Katarina Truvé, Helene Sjögren, Sándor Dósa, Magnus Tisell, Birgitta Lannering, Fredrik Enlund, Tommy Martinsson, Pierre Åman, Frida Abel

In the Data Availability Statement, the GenBank ID is listed incorrectly. The correct statement is: The novel cDNA and protein sequence of the GTF2I-BRAF 19–10 fusion gene has been deposited to GenBank (BankIt) at NCBI under Accession Number KY884638.
