# Supplementary material for: A new GTF2I-BRAF fusion mediating MAPK pathway activation in pilocytic astrocytoma
Source: PLoS One. 2017 Apr 27;12(4):e0175638. doi: 10.1371/journal.pone.0175638 (PMC5407815; doi:10.1371/journal.pone.0175638)
Supplement: S1 Fig — (PDF) [file pone.0175638.s001.pdf]

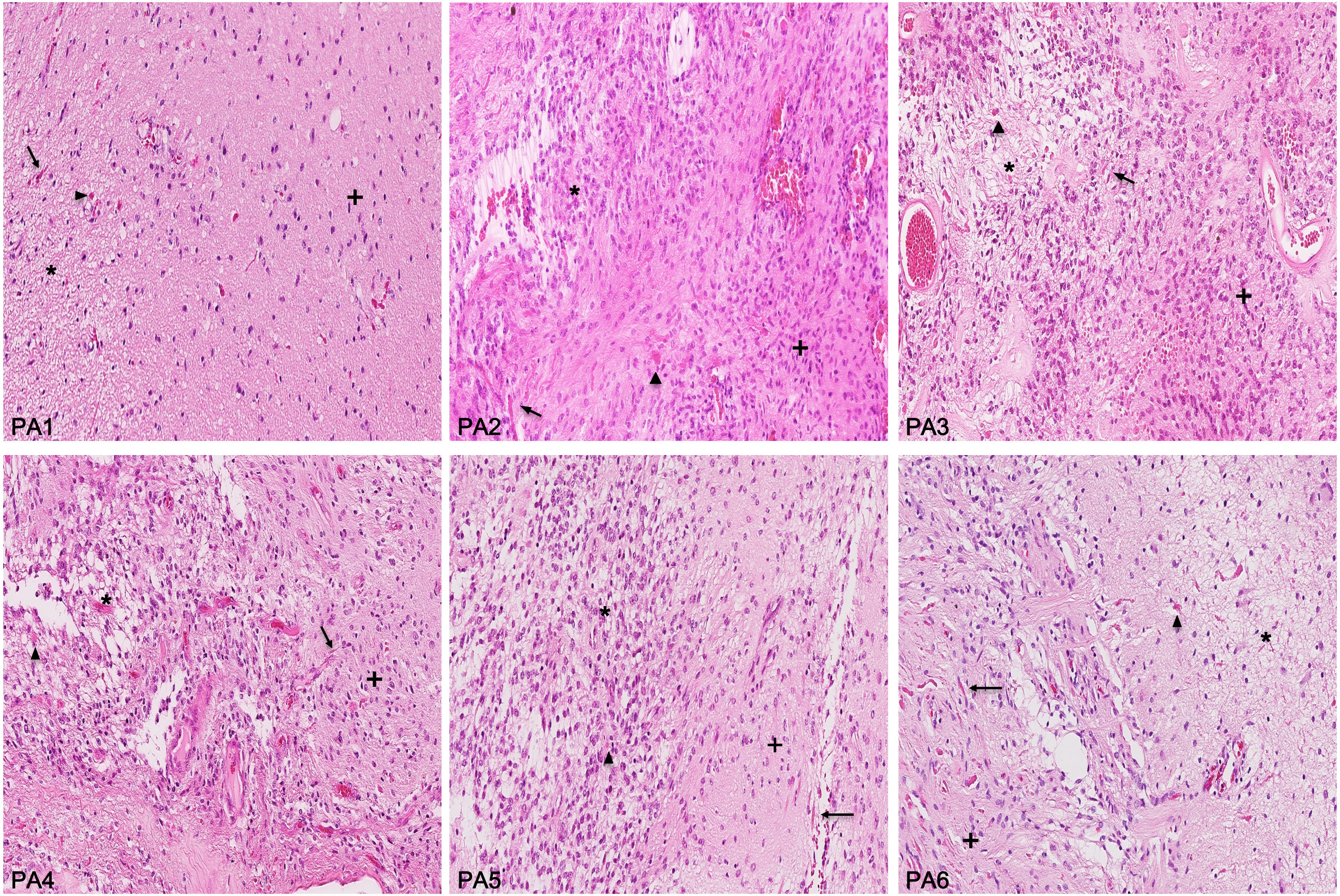

S1 Fig. Hematoxylin-Eosin staining of six PA cases. PA histopathological characteristics; compact (+) and loose (*****) areas, Rosenthal fibers (arrow), eosinophilic granular bodies (arrow head). 200x magnification.
