# Supplementary material for: A new GTF2I-BRAF fusion mediating MAPK pathway activation in pilocytic astrocytoma
Source: PLoS One. 2017 Apr 27;12(4):e0175638. doi: 10.1371/journal.pone.0175638 (PMC5407815; doi:10.1371/journal.pone.0175638)
Supplement: S2 Fig — (PDF) [file pone.0175638.s002.pdf]

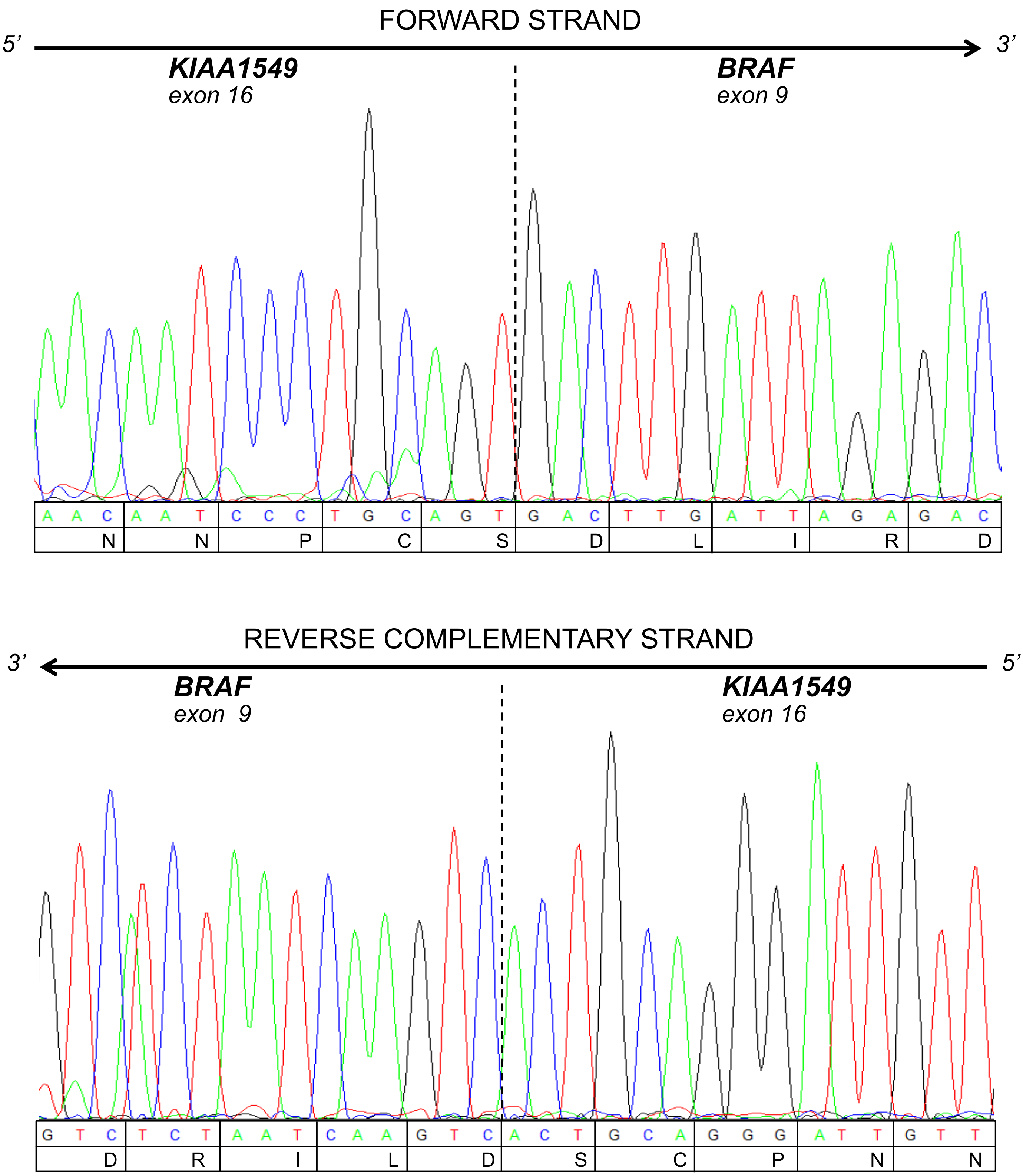

S2 Fig. Sanger sequencing of the RT-PCR product (249bp) generated from the KIAA1549-BRAF 16-9 fusion of in case PA6. The electropherogram display 15bp spanning the junction, and translation into amino acids (aa). The KIAA1549-BRAF exon 16-9 fusion generates an in-frame junction and a putative fusion protein of 2135 amino acids (aa).
