# Supplementary material for: A new GTF2I-BRAF fusion mediating MAPK pathway activation in pilocytic astrocytoma
Source: PLoS One. 2017 Apr 27;12(4):e0175638. doi: 10.1371/journal.pone.0175638 (PMC5407815; doi:10.1371/journal.pone.0175638)
Supplement: S3 Fig — (PDF) [file pone.0175638.s003.pdf]

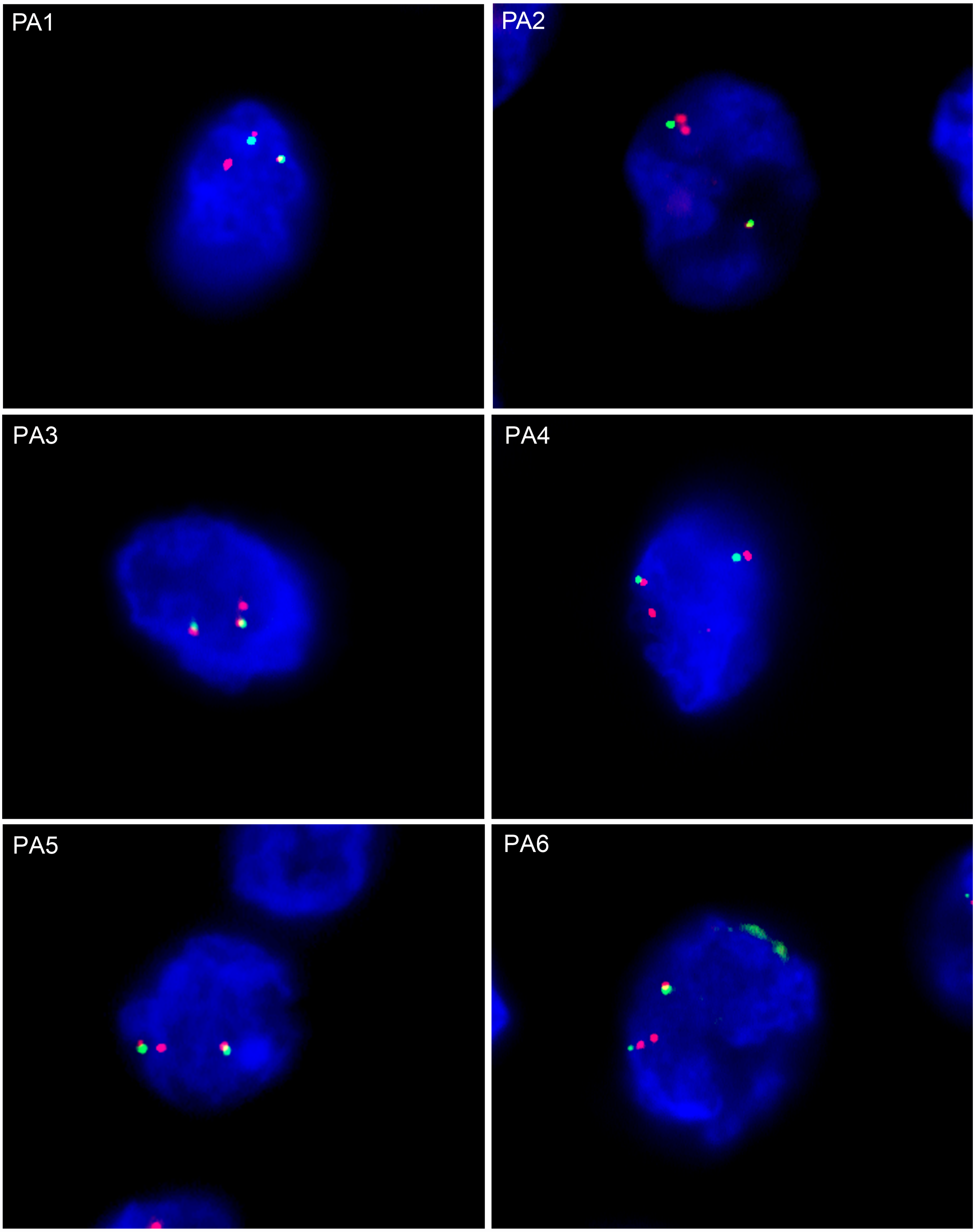

S3 Fig. *BRAF*-fusion detection with *BRAF* Break Apart FISH in six PA cases.The *BRAF* break apart pattern was characterized in six PA cases harboring two different *BRAF*-fusions (*GTF2I-BRAF* in PA3; *KIAA1549-BRAF* in PA1-2 and PA4-6). In fusion-positive nuclei, the interphase FISH displayed two pairs of merged (yellow) or adjacent green/red signals of the 5'/3'wt BRAF alleles, and one additional split red signal indicating a duplicated copy of the 3' BRAF region. All six BRAF-fusion-positive cases showed the break apart pattern, although the distance of the split 3' signal from the normal BRAF allele pairs differed between cases; close split signal (*e.g.* PA2, PA5) or distant split signal (*e.g.* PA4) from the normal allele pairs in 7q34.
